# Supplementary figures and images for: Impact of social class on health: The mediating role of health self-management
Source: PLoS One. 2021 Jul 16;16(7):e0254692. doi: 10.1371/journal.pone.0254692 (PMC8284807; doi:10.1371/journal.pone.0254692)

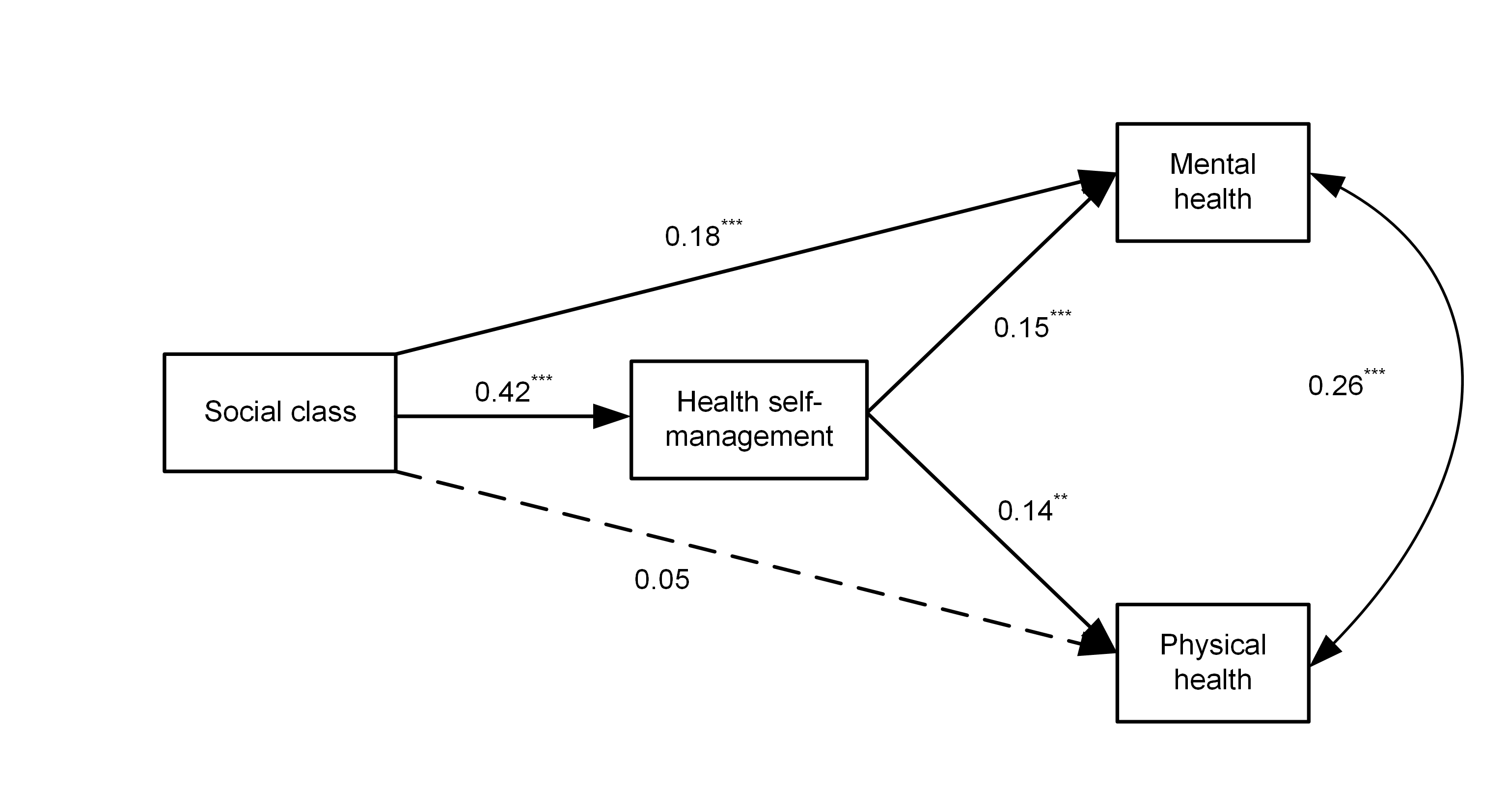

Supplement: S1 Fig — Age are controlled for but are not illustrated for simplicity. **p < .01, ***p < .001. (TIF) [file pone.0254692.s001.tif]
